# Generation and Characterization of the *Drosophila melanogaster paralytic* Gene Knock-Out as a Model for Dravet Syndrome

**DOI:** 10.3390/life11111261

**Published:** 2021-11-18

**Authors:** Andrea Tapia, Carlo N. Giachello, Martina Palomino-Schätzlein, Richard A. Baines, Máximo Ibo Galindo

**Affiliations:** 1 Centro de Investigación Príncipe Felipe, 46012 Valencia, Spain; atapia@cipf.es (A.T.); mpalomino@cipf.es (M.P.-S.); 2 Manchester Academic Health Science Centre, Division of Neuroscience and Experimental Psychology, School of Biological Sciences, Faculty of Biology, Medicine and Health, University of Manchester, Manchester M13 9PL, UK; carlogiachello.cg@gmail.com (C.N.G.); Richard.Baines@manchester.ac.uk (R.A.B.); 3 Instituto Interuniversitario de Investigación de Reconocimiento Molecular y Desarrollo Tecnológico (IDM), Universitat Politècnica de València, Universitat de València, 46022 Valencia, Spain; 4 UPV-CIPF Joint Unit Disease Mechanisms and Nanomedicine, 46012 Valencia, Spain

**Keywords:** Dravet syndrome, epilepsy, voltage-gated sodium channel, gabaergic neurons, electrophysiology, metabolomics

## Abstract

Dravet syndrome is a severe rare epileptic disease caused by mutations in the *SCN1A* gene coding for the Nav1.1 protein, a voltage-gated sodium channel alpha subunit. We have made a knock-out of the *paralytic* gene, the single *Drosophila melanogaster* gene encoding this type of protein, by homologous recombination. These flies showed a heat-induced seizing phenotype, and sudden death in long term seizures. In addition to seizures, neuromuscular alterations were observed in climbing, flight, and walking tests. Moreover, they also manifested some cognitive alterations, such as anxiety and problems in learning. Electrophysiological analyses from larval motor neurons showed a decrease in cell capacitance and membrane excitability, while persistent sodium current increased. To detect alterations in metabolism, we performed an NMR metabolomic profiling of heads, which revealed higher levels in some amino acids, succinate, and lactate; and also an increase in the abundance of GABA, which is the main neurotransmitter implicated in Dravet syndrome. All these changes in the *paralytic* knock-out flies indicate that this is a good model for epilepsy and specifically for Dravet syndrome. This model could be a new tool to understand the pathophysiology of the disease and to find biomarkers, genetic modifiers and new treatments.

## 1. Introduction

### 1.1. Dravet Syndrome

Dravet syndrome (DS, ORPHA:33069), or epileptic encephalopathy with early onset 6 (EIEE6), or severe childhood myoclonic epilepsy (SMEI), is a childhood epilepsy [1,2,3]. DS has an early onset in the first few months of life, usually triggered by fever, with a hemiclonic or clonic seizure, affecting one or both sides of the body, respectively, which is followed by further episodes that can be prolonged and even result in *status epilepticus* (SE, duration over 5 min or repeated short episodes). With time, patients usually suffer more febrile or afebrile seizures that can be of different types from the initial ones: tonic-clonic, myoclonic, absence. In addition to seizures, they can also suffer cognitive delay, movement disorders and, more seriously, sudden death in a relatively high proportion (>10%). DS is a disease with a very low prevalence, it is estimated that one infant suffers it for every 20,000 to 40,000 births. In 80% of cases, this pathology is caused by the mutation in one of the *SCN1A* gene alleles [4]. The *SCN1A* gene codes for the Nav1.1 protein, which is the α subunit of the type I voltage-gated sodium channel. In 95% of cases the pathological mutations in *SCN1A* are de novo, there are no family members with the same mutation. It is assumed that the disease is caused by haploinsufficiency, that is, the expression of half of the protein encoded by *SCN1A* is not sufficient for the proper functioning of neurons that express the channel. When the genetic basis of DS was uncovered [5], it came as a surprise since voltage-gated sodium channels are usually involved in the excitation of neuronal and muscular cells. The conundrum was resolved when it was found that the mutations primarily prevented the activation of GABAergic inhibitory interneurons, and not so much excitatory neurons, thus disrupting the excitation/inhibition balance [6,7].

In DS and similar epileptic syndromes, seizures result in damage of neural tissue through oxidative stress, inflammation, and/or metabolic imbalance [8,9]. NMR metabolomics of serum samples of paediatric drug-resistant epilepsy patients revealed alterations in metabolites related to inflammation and oxidative stress, such as increased N-acetyl-glycoproteins, lactate, creatine, glycine and lipids or decreased citrate [10]. These metabolites could be used as biomarkers to study disease progression, or to evaluate the effect of pharmacological interventions.

The first-line treatment for epilepsy is anti-seizure drugs (ASDs). However, in spite of the availability of many ASDs, approximately one-third of patients fail to achieve seizure control or soon become resistant to the effects of the ASDs [11]. In DS, there are no efficient medications to control seizures and the neurological damage, although some first line ASDs, such as valproate, stiripentol, or benzodiacepines have shown limited effectiveness [12]. The most recent therapeutic development has been the successful clinical trials of cannabidiol [13], which was approved to treat DS in America and Europe in 2019.

Although the majority of DS patients have mutations in the *SCN1A* gene, and these are always dominant, each mutation produces a different clinical picture and responds differently to anticonvulsant treatments [14]. Further proof of the complexity of the genotype-phenotype correlation of *SCN1A* mutations is that, in addition to DS, these mutations can cause a spectrum of epileptic neuropathy disorders. Given this picture, it is impossible to develop a single disease model that can be used to understand the disease mechanisms and to find new therapies for all the mutations known. The most effective approach to this would be the development of precision medicine models reproducing the genotypes of a wide range of mutations in *SCN1A*. At this time there are no curative treatments for DS, only palliative ones.

### 1.2. The Paralytic Gene of Drosophila

In rare diseases such as DS, with a low prevalence and a lack of therapeutic options, the International Rare Diseases Research Consortium (IRDiRC) guidelines recommend the use of non-mammalian disease models for a more efficient research [15]. *Drosophila melanogaster* has a gene, *paralytic* (*para*), which is equivalent to all the human genes encoding voltage-gated sodium channels (Nav1.X). In fact, mutations in *para* were known to cause seizures a decade before the discovery of the molecular basis of DS [16]. Mutations in this gene are associated with epileptic episodes similar to those that occur in patients [17]. Flies mutant for the *para^bss1^* allele, a bang-sensitive mutation, display a behaviour very similar to wild type flies under normal conditions, and alterations are only observed when exposed to a mechanical shock by vortexing for a few seconds. Crises show six different phases in adult flies and have an average duration of 240 s. (1) The initial crisis lasts several seconds and manifests with shaking of the legs, abdominal contractions, flutter and extension of the proboscis. (2) In the early state of paralysis: flies are immobile and without response to external stimuli. (3) The period of tonic-clonic crisis, the fly remains motionless most of the time (tonic phase) but intermittently suffers from generalised shaking (clonic phase). (4) The recovery crisis, at this stage, the fly again presents movements similar to the initial crisis mixed with clonic activity. (5) In the refractory period flies begin to behave normally but further crises cannot be induced by any external stimulus, unless stimulus strength is greatly increased. (6) After recovery the fly has exceeded the refractory state and by this time they can be stimulated again to present crises. Seizures can be triggered by different stimuli: mechanical (agitation), thermal, visual, or electrical.

The Para protein sequence is considerably conserved, such that a majority of clinical mutations due to non-synonymous changes fall back on amino acids that are identical or similar. Because of these similarities, *Drosophila* has been used in studies to understand the mechanism of disease mutations in *SCN1A* [18]. As a result of its remarkable features as a model organism, *Drosophila* has also been used to find genetic and pharmacological modifiers of the *para* seizure phenotype, which can be directly relevant for DS [19].

Our overarching objective is to replace the *Drosophila para* gene with the human *SCN1A* gene harbouring mutations representing the clinical spectrum. For this, we are using a technique that has been specifically developed for gene replacement in *Drosophila* [20]. The first step in this strategy is the knock-out of the *para* gene by inserting a construct that interrupts the expression of the gene, and also has a *phiC31* phage *attP* site that can be used to insert human cDNAs. This initial step is interesting on its own since, if successful, it is expected to generate a null allele of the *para* gene due to the absence of a functional transcript, which in itself is a useful tool to understand the convulsive phenotype, but also can help us in the search for new treatments for DS.

## 2. Materials and Methods

### 2.1. Molecular Biology

To generate the gene-targeting construct, two arms homology were designed. They flank the start codon in the first coding exon and successful recombination would generate a small deletion of 12 bp. The sequences of the primers that we used to generate the homology arms, and all the subsequent molecular biology analyses, are indicated in Table A1. After high fidelity PCR with Pfu DNA polymerase (Biotools; Madrid, Spain) using genomic DNA of the wildtype *Canton-S* strain as a template, the fragments were digested with the appropriate restriction enzymes (Thermo-Fisher Scientific, Waltham, MA, USA) and cloned in the corresponding multiple cloning site of the pTV[Cherry] plasmid [20]. The resulting plasmid sequence was tested by Sanger sequencing.

For quantitative PCR, total RNA was extracted from pools of 15–20 fly heads or whole larvae with Trizol (Thermo-Fisher Scientific, Waltham, MA, USA). To generate the cDNA we used the qScript cDNA SuperMix kit (Quantabio, Beverly, MA, USA). The quantitative PCR was performed with SYBR Green Master Mix (Applied Biosystems, Foster City, CA, USA) using a LightCycler 480 (Roche, Bassel, Switzerland). All the samples were analysed in triplicate and transcript levels were calculated by the 2^−ΔΔ*C*t^ method, with normalisation by comparing to expression of the *rp49* reference gene.

### 2.2. Generation of the para^KO^ Allele

We used a homology-directed recombination technique developed by Baena-López et al., which comprises two steps [20]. First, we obtained a stock containing de donor construct inserted in the genome by *P* element transposase, since the donor plasmid is flanked by the *P* element inverted repeats. The transgenics were generated by the *Drosophila* transgenics service at Centro de Biología Molecular Severo Ochoa in Madrid, and were selected by the red eye colour due to the *mini-white* reporter within the construct. Since we were going to target the first chromosome, we chose an insertion in the third chromosome to facilitate the process. We crossed individuals carrying the donor construct to *hs-FLP*, *hs-SceI* flies and activated the heat shock promoter by incubation for 1 h at 37 °C twice, during the second and third larval instars, to induce the excision and the linearisation of the construct. In the progeny, we selected females that had mosaic eyes, and crossed them to *Ubi-Gal4* males, so that Gal4-directed expression of *reaper*, which is outside the arms of homology, would kill the cells where the construct had not excised or had re-inserted non-specifically. After scoring 15,000 flies, we selected 3 candidates by the presence of the eye colour marker, and one of them mapped to the first chromosome. The correct insertion was validated by PCR using different primer pairs (Table A1). We also generated a stock from a white-eyed sibling to be used as a control, since it would have the same genetic background as the *para^KO^*, except for the gene editing of the *para* gene. To remove most of the insert, leaving only a minimal insertion with an *attP* site, *para^KO^*/*FM7c* females were crossed to *hs-Cre* males and the progeny was heat shocked twice in second and third instar stages. Excision candidates were selected by the white eye colour, and validated by PCR with flanking primers and sequencing of the PCR product.

### 2.3. Drosophila Husbandry and Genetics

All the strains used in this work and their origin are listed in Table A2. The flies were cultured at 25 °C in standard cornmeal medium. Since the *para^KO^* allele is homozygous/hemizygous lethal, it was kept over an *FM7c* balancer chromosome.

To determine the stage of lethality, and to select homozygous embryos before they died for RNA extraction, we used the *FM7c-GFP* balancer chromosome. Parental flies were allowed to mate in a standard vial and then transferred to egg-laying plates containing 25% apple juice and 2.5% agar supplemented with fresh yeast paste. Adults were transferred to a fresh plate after 6 h, and embryos were allowed to age for a further 12 h, and selected according to the presence or absence of GFP fluorescence. For RNA extraction, the embryos were collected in tubes and frozen at −80 °C until the time of extraction. To determine the stage of death, GFP-negative embryos were allowed to age and then examined at the microscope.

### 2.4. Seizure Phenotypes

Seizure phenotypes were characterised as previously described [21]. For the mechanically-induced seizure assay, 10–15 flies were transferred to an empty vial and left to settle for 30 min, then they were vortexed at top power for 10 s. After this we determined how many flies have gone into seizure and how long they took to recover. The experiments were performed in triplicate.

For the temperature induced seizures, the flies were transferred to vials and left to settle as above, and then submerged in a water bath at 40 °C for 2 min and recorded with a video camera. The video was then analysed to determine how many flies went into seizure, and at which time they did so. In this experiment, recovery is almost immediate after cooling down, so it is not a relevant parameter. The experiments were also performed with 10–15 flies in triplicate.

In order to simulate the sudden death, usually associated with *status epilepticus* (SE), we performed longer incubations at 37.5 °C, which is not so effective at causing seizures. Vials were submerged in the water bath, incubated for 30 min and then checked that some individuals had started to seize. Then, the vials were incubated for a further 15 min, and the number of flies that had died was scored. Sample size and replicate number was as in the previous experiments.

The seizure assay in third instar larvae was performed following a published protocol [19]. In total, 30 larvae per genotype were placed on a Petri dish, and electroshocked with a probe composed of two tungsten filaments, 0.1 mm in diameter and 1.2 mm of separation between them. The shock was applied in the anterior dorsal part of the larvae, just above the central nervous system, by a 3 s pulse at 30 V generated by a Grass S88 stimulator (Grass instruments, West Warwick, Rhode Island). For each larva, we scored the recovery time, which is defined as the time it takes to resume a normal movement pattern after the seizure, usually manifested as paralysis and tonic contractions.

### 2.5. Negative Geotaxis

The climbing experiments were carried out following standard procedures [22]. A total of 10–15 flies were transferred to empty vials and left to settle for 30 min. A line was drawn with a permanent marker along the circumference of the vial at a distance of 80 mm from the base. The vials were then hit against a cork pad to knock down the flies to the bottom and the flies were video recorded while they attempted to climb back to the top. The video was analysed to determine which proportion of flies had managed to cross the 80 mm mark after 10 s. The experiments were performed in triplicate, and each replica was assayed 5 times, allowing half an hour between experiments.

### 2.6. Flight Assay

The method was adapted from Babcock and Ganetzky [23]. 30–40 the flies were introduced in an empty vial and left to settle for 30 min. We built a methacrylate flight tester tube, 90 cm high and 12 cm in diameter, with an interior PVC plastic sheet coated with rodent trap adhesive glue (Cofan, Ciudad Real, Spain), such as the flies were glued to the cylinder wall at variable height dependent on their ability to stabilise their flight. A ‘drop tube’ was added to ensure that flies entered the flight tester at the same speed, thus reducing variability associated with manipulation. The flies were dropped into the tester and then the plastic sheet was removed and photographed. The images were analysed using the ImageJ 1.53c software (Rasband, W.S., ImageJ, U. S. National Institutes of Health, Bethesda, MD, USA, https://imagej.nih.gov/ij/ 16 November 2021, 1997–2018). We performed three replicates for each experiment, with a total of 80 flies per genotype.

### 2.7. Locomotion Assay

The method was adapted from Stone et al. [24]. Individual flies were placed in a 90 mm petri dish resting on a white LED light source covered with a paper sheet. The fly was recorded by a zenithal video camera for 10 min using the VirtualDub 1.10 software (a free and open-source video capture and processing utility written by Avery Lee and licensed under the GNU General Public License; https://www.virtualdub.org 16 November 2021) at a speed of 2 photograms per second. The individual photograms were uploaded in ImageJ, and the contrast adjusted to facilitate the identification of the object of interest (individual fly) to generate a file with the coordinates of the fly in each photogram. Then, the coordinates table was introduced in an Excel spreadsheet to calculate the total distance and the average speed. In addition to this, an internal area, 50 mm in diameter and concentric with the larger arena, was defined in order to determine the proportion of time spent in the central and peripheral regions of the arena. For each genotype, 10 individual flies were assayed in triplicate.

### 2.8. Learning and Memory

These parameters were assessed by the aversive phototaxic suppression assay [22]. Flies have positive phototaxis, they tend to move towards a source of light. We constructed a T-maze consisting of two transparent plastic vials, one of them darkened with aluminium foil, separated by a sliding door. Flies were tested individually, after 16 h fasting with only a water-soaked paper to avoid dehydration. Each fly was placed in the darkened vial with the door closed, and left to habituate for a few minutes. After this, the door was opened and a strong light shined next to the other vial, which also contained a filter paper soaked with 120 µL of 0.1 M quinine (Sigma-Aldrich, Saint Louis, MO, USA) as an aversive stimulus that should oppose the phototaxic attraction. Each training session lasted one minute and was performed six consecutive times. Next, five testing session were performed, scoring whether or not the fly crossed the barrier in the 10 s following the opening of the door. To assess the short term memory, the same flies were tested again after five hours. The learning and memory tests were performed in 30 flies of each genotype, and in triplicate.

### 2.9. Electrophysiology

Patch clamp recordings from the aCC motoneuron were performed as previously described [25]. For both voltage clamp and current clamp experiments, the electrodes were made using borosilicate glass capillary tubes (GC100F-10, Harvard Apparatus, Edenbridge, UK) pulled with a P-1000 capillary puller (Sutter Instrument, Novato, CA, USA) and then fire-polished to a resistance between 10–15 MΩ.

Signals were recorded using a Multiclamp 700B amplifier linked to a Digidata 1440A controlled by pCLAMP (version 10.2, Molecular Devices; Sunnyvale, CA, USA). Recordings were filtered at 10 Hz and sampled at 20 KHz. Recordings of Na^+^ currents were performed using an on-line P4 leak subtraction protocol. Voltage steps were applied from a prepulse holding potential of −90 mV. Only cells with an input resistance of >500 MΩ were accepted for analysis. Cell capacitance was calculated by integrating the area under the capacity transients elicited by stepping the cell from −60 to −90 mV for 30 ms.

### 2.10. Metabolomics

Metabolite extraction, spectral recording and data analysis were performed as previously described [26]. Briefly, for metabolite extraction 20–23 freshly dissected fly heads were homogenised and extracted with the chloroform-methanol-water extraction procedure to separate aqueous and organic extracts, which were lyophilised and kept at −80 °C. Aqueous extracts were resuspended in 0.1 M phosphate buffer made with deuterated water, and organic extracts were resuspended in deuterated chloroform. Then, 3-(Trimethylsilyl) propionic-2,2,3,3-d4 acid sodium salt (TSP) and Tetramethylsilane (TMS) were used as internal standards for the aqueous and the organic phase, respectively. ^1^H NMR spectra of the extracts were recorded at 27 °C on a Bruker AVII-600 spectrometer (Bruker Scientific Instruments, Billerica, MA, USA) using a 5-mm TCI cryoprobe, with 512 scans. Water presaturation was applied for aqueous samples to minimise the water signal. In total, six independent replicates containing 20–23 individuals each, from two independent experiments were analysed.

After acquisition, spectra were processed with an exponential line broadening factor of 0.5 and automatic phase correction performed. Spectra were then referenced to the signal of the internal standards (TSP/TMS) at 0 ppm. The signals were assigned to the corresponding metabolites with the help of our previous work [24] and data bases Human Metabolome Database (HMBD) [27] and BioMagResBank (BMRB) [28]. Spiking experiments with external standards were performed to confirm the assignment of glucose, threonine, taurine, and betaine. Spectra were integrated automatically with MestreNova 12 with the GSD deconvolution option. Integration tables were normalised to total intensity for multivariate data analysis, we used SIMCAP 14.1 (Umetrics, Umeå, Sweden). Principal components analysis (PCA) was used as an unsupervised model after univariate scaling of the data. To assess the quality of the each model, the parameters R2 (goodness of fit) and Q2 (goodness of prediction) were evaluated. A Q2 value ≥ 0.5 was considered indicative of a good model.

### 2.11. Statistical Analysis

In all the experiments, sample size and number of replicates were determined with the help of the available literature to balance the statistical power required and the experimental complexity. The sample size of the control and experimental genotypes was always identical or highly similar.

In all the experiments, we applied the Kolmogorov–Smirnov test for normal distribution. In pairwise comparisons between the experimental and control groups, we applied the T-test if the distribution was normal, and the non-parametric Mann–Whitney test if they were not normal. In the experiments, involving more than two groups or more than one variable (variation of the amplitude of sodium currents at different membrane potentials, learning and memory), if the distributions were normal we used the one-way or two-way ANOVA, followed by Bonferroni post hoc test; if they were not normal we used the non-parametric Kruskal–Wallis test. In the lifespan experiments we used the Mantel–Cox test.

The GraphPad Prism software (GraphPad Software, San Diego, CA, USA) was used for illustrations and statistics. In scatter plot diagrams, the displayed data are the mean and the standard deviation.

## 3. Results and Discussion

### 3.1. Generation and Validation of the para Knock-Out

**Figure 1:**
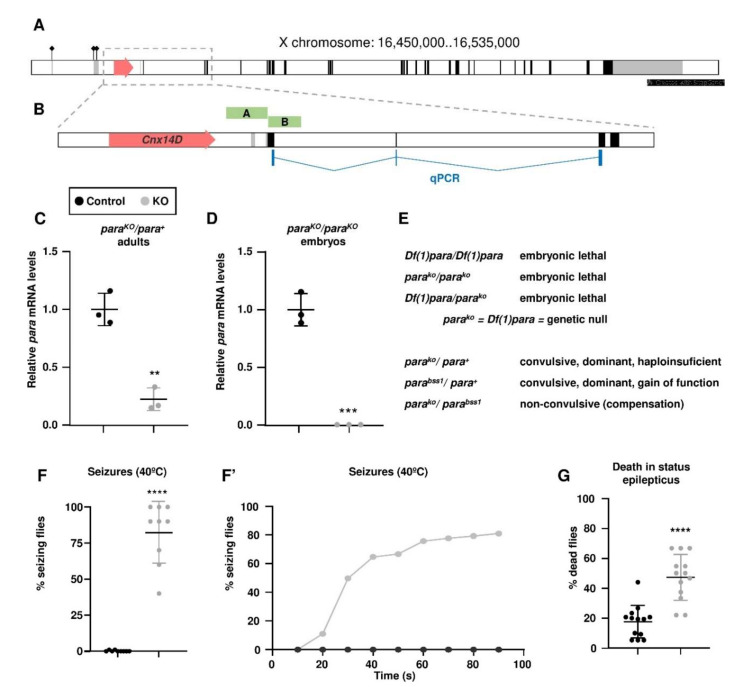
Generation and validation of the *para* knock-out. (**A**) Structure of the para locus, filled arrows represent transcribed regions, the coding and non-coding regions are shaded black and grey respectively; the flags indicate the three alternative start of transcription sites; the red arrow represents the *Calnexin 14D* gene nested within a *para* intron. (**B**) Magnification of the boxed area around the start of transcription: the green boxes indicate the extension of the A and B arms of homology flanking the start codon; the sequence amplified in the qPCR to estimate *para* mRNA levels is indicated in blue. (**C**,**D**) relative *para* mRNA levels in control versus *para^KO^*/*para^+^* heterozygote females (**C**) and in control versus *para^KO^* hemizygote/homozygote embryos (**D**) estimated by quantitative PCR. (**E**) Genetics of the *para* locus regarding embryonic lethality and convulsive phenotype. (**F**,**F’**) proportion of flies undergoing seizures at 40 °C displayed as total number of flies at the end of the experiment (**F**) and as temporal dynamics of the seizures (**F’**). (**G**) Estimation of deaths in status epilepticus by exposure to a moderate heat shock at 37.5 °C for 15 min. (**C**,**D**,**G**) Student’s *t*-test; (**F**) Mann-Whitney. Bars represent mean ± SD (** *p* < 0.01; *** *p* < 0.005; **** *p* < 0.0001).

The *para* gene is one of the largest and most complex in the *Drosophila* genome, spanning nearly 80 kb and with several isoforms generated by alternative splicing and start of transcription (Figure 1A). A second gene, *Calnexin 14D*, is nested within the first intron of *para*. To design the knock-out strategy we downloaded the locus sequence, corresponding to bases 16,450,000 to 16,535,000 of the X chromosome reference sequence (NC_004354.4). We designed two arms of homology, an A arm of 951 bp ending in the base pair just upstream of the start codon, and a B arm of 782 bp, starting 10 bp downstream of the start codon (Figure 1B), and both arms were cloned into the *pTV[Cherry]* vector to achieve the gene editing. Due to the design of the strategy, the homologous recombination would generate a deletion of 12 bp, including the start codon, followed by the insertion of the construct, which would prevent the translation of the full length protein. After the generation of the transgenic flies and the induction of the homologous recombination, we selected a candidate knock-out insertion. The construct carries the mCherry reporter gene with a minimal promoter that normally reproduces the pattern of expression of the edited gene; in this case the first indication that our editing had been correct was the presence of red fluorescence in the central nervous system (not shown). For a thorough validation of the insertion, we performed two PCRs at either end of the insertion, with one primer designed from the transgene sequence and a second primer that would hybridise in the flanking genomic sequence, beyond the sequence used to generate each one of the arms of homology, to discard non-specific insertions. Both PCRs were successful, and the products were sequenced to check that the sequence was the expected from a legitimate recombination.

After this, we removed most of the cassette by crossing to flies carrying it to flies expressing the Cre recombinase, leaving just a minimal *attP* insertion and the 12-bp deletion. The excision was also validated by PCR with primers designed from the genomic region flanking the insertion and sequencing. For all the subsequent experiments, we compared the *para^KO^* flies to a control genotype. Since the new allele was generated in a *w^1118^* background, and this can affect viability and fitness, the experimental genotype was an outcross of the *para^KO^* stock to wild type *Canton-S*, from which the *para^KO^*/*para^+^* females were selected. For the control, we used a stock generated from the siblings of fly used to make the *para^KO^* stock. These flies had gone through the same genetic crosses, but the gene editing construct had failed to recombine. These flies were also crossed to *Canton-S* to obtain the control individuals.

Both the original and the minimal insertions were homozygous lethal in females and hemizygous lethal in males, so we kept the mutation over an *FM7c* X-chromosome balancer. All the males in the stock were *FM7c*/*Y*, and the females were *para^KO^*/*FM7c*. In order to determine the period of lethality, we put the *para^KO^* allele over an *FM7c-GFP* balancer, which expresses GFP under the pattern of the *Kruppel* gene. GFP-negative embryos were able to complete development, displayed normal cuticular structures, but were unable to hatch. Therefore, lethality was most probably due to impairment of the neural function.

Gene editing and deletion of the first four codons should prevent protein translation, but since the next in-frame start codon is the fourth exon several kilobases downstream, it was possible that the mutant transcript was unstable due to nonsense-mediated mRNA decay. To test this possibility we designed a quantitative PCR primer pair which should amplify from the end of the first coding exon up to the third exon, both in regions downstream of the edited sequence. We performed the quantitative PCR in heterozygous adult female head extracts, and we observed a marked reduction in the *para* transcript levels (Figure 1C). In addition, we also quantitated transcription in homozygous/hemizygous embryos selected by the absence of GFP fluorescence, in this case the mRNA was virtually absent (Figure 1D). Expression of the control gene *rp49* was unchanged. These results confirm that the editing of the *para* gene destabilises the transcript and results in a full loss of function of the locus.

Regarding the genetics of the allele, in addition to recessive embryonic lethal, it is dominant regarding seizure susceptibility, as expected (Figure 1E). The heterozygous females displayed a convulsive phenotype upon heat shock, but not by mechanical stress caused by vortexing (see below). Since the mutation abolishes gene expression, the most likely explanation is that it is haploinsufficient. Another allele, *para^bss1^*, has been described as dominant but in this case as the result of a gain of function [29]. In agreement with this, the *para^bss1^*/*para^KO^* heterozygote was not convulsive due to the compensation of loss and gain of function effects.

Temperature-induced seizures are the first manifestation of DS, and these are followed by further febrile or afebrile seizures, which are the most disabling and life-threatening features in DS patients [1]. This phenotype is also present in preclinical models of DS [30]. Consistent with this, the most evident convulsive phenotype of *para^KO^* flies was obtained by heat shock, submerging the vial in a 40 °C water bath (Figure 1F). In these conditions, there was no effect on control females flies, but around 80% of heterozygote females suffered seizures; most of the time they laid on their back, paralyzed, and suffered occasional spasms. When we looked at the temporal pattern, the first flies started to seize as early as 20 s after being submerged, and in one minute they approached the maximum level of 80% (Figure 1F’). The paralyzed flies resumed their normal behaviour immediately after removing the vials from the water bath, which was already described in other Drosophila models of DS [18].

Another problem associated with the disease is a considerably high mortality rate, mostly caused by SUDEP or by SE [31]. Regarding SUDEP, control and *para^KO^* flies did not show any difference in survival, therefore there was no SUDEP-like phenotype in our model. In contrast, most of the mouse models of DS have a high mortality rate [6,32,33,34,35,36,37]. This is probably due to the fact that *para^KO^* flies did not have spontaneous seizure activity when reared in normal conditions, unlike the different mouse models. In order to generate a condition similar to the SE, an experiment was designed in which the flies subject to a longer stimulus, by keeping them 45 min in a hot water bath at 37.5 °C, and then scored the number of flies that had not survived this condition. There was a statistically significant increase of around three-fold in death in the *para^KO^* flies (Figure 1G). Therefore, a maintained seizure state similar to SE did affect the *para^KO^* heterozygote females differentially, regarding lethality.

### 3.2. The para^KO^ Flies Suffer Additional Neuromuscular and Cognitive Alterations

**Figure 2:**
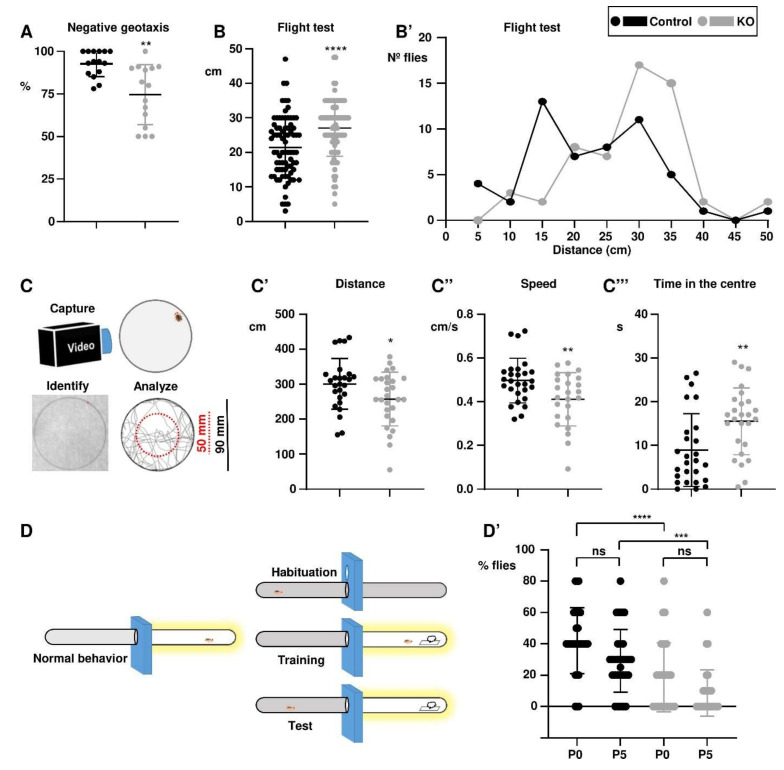
Neuromuscular and behavioural analyses. (**A**) Negative geotaxis assay, proportion of the flies that reached the 8 cm mark in 10 s. (**B**,**B’**) Flight test, indicating the average distance of stabilisation of the control and *para^KO^* flies (**B**), as well as the distribution of the distances reached in each genotype (**B’**). (**C**–**C’’’**) For the locomotion assay, individual flies were filmed for 10 min in a closed arena 90 mm in diameter, and the resulting video was analysed to extract the relevant parameters (**C**); these included total distance walked (**C’**), average speed (**C’’**) and time spent in an internal area of 50 mm. (**D**,**D’**) For the learning and memory assays, individual flies were subject to an aversive phototaxic suppression assay (**D**) at two different times, immediately after training (P0) and after 5 h (P5) to evaluate each one of these parameters (**D’**). (**A**,**B**) Mann-Whitney test; (**C**–**C’’**) Student’s *t*-test < (**D’**) one-way ANOVA plus Kruskal–Wallis. Bars represent mean ± SD (* *p* < 0.05; ** *p* < 0.01; *** *p* < 0.005; **** *p* < 0.0001).

Although seizures are the most disabling and life-threatening feature in DS patients, they suffer from additional impairments, such as movement disorders, learning difficulty, and cognitive and behavioural impairment [1,38,39,40]. These alterations are also present in available zebrafish and mouse models [30,41]. In order to evaluate the neuromuscular fitness of the *para^KO^* flies, we performed tests for negative geotaxis and flight. In the negative geotaxis test, flies are knocked down to the bottom of the vial, and then scored for their ability to climb back to the top. In this test the performance of the *para^KO^* flies was slightly worse than for the control flies (Figure 2A). The flight test measures the ability of individual flies to stabilise their flight and reach the wall after being dropped into a cylinder. In this case, the *para^KO^* flies also took longer to stabilise than control flies (Figure 2B). Looking at the performance in more detail, control flies tended reach the wall 15 to 30 cm from the drop point, while most *para^KO^* flies stabilised at 30–35 cm (Figure 2B’). Overall, the *para^KO^* flies had worsened performance in climbing and flight tests.

Another test that can be used to analyse more complex behaviours is a locomotion assay in a closed arena. The intensity and patterns of locomotion are related to motility and to the degree of anxiety of the fly (Mohammad et al., 2016), similar to mice and other animals: flies in an enclosed space tend to remain close to the walls for safety. In this case, individual flies were allowed to explore a circular arena 90 mm in diameter, and were filmed during 10 min; and then image analysis software was used to extract motility parameters (Figure 2C). Compared to the control flies, the *para^KO^* flies walked a shorter distance and did so at a lower average speed (Figure 2C,C’). This was a quantitative difference that could be equivalent to the performance in the negative geotaxis and flight tests, just reflecting a worse neuromuscular performance. A third analysis, the time spent in the centre of the arena, reflects a more complex behaviour, the pattern of movement; which can depend on decision such as the choice between exploratory or protective paths. In this case, the *para^KO^* flies spent more time in an internal area, 50 mm in diameter. Anxiety is one of the comorbidities in DS patients [42], and similar anxiety phenotypes have been described in open field experiments with mouse models [37].

*Drosophila* has positive phototaxis, and this behaviour can be used to test learning and short term memory by exposure to a negative stimulus (Figure 1D). To this end, individual flies were left to habituate to the system inside a darkened chamber, which was then connected to an illuminated chamber to attract them. Following their phototaxic impulse they tended to move into the illuminated chamber, but there they found a filter paper soaked in quinine, which has a bitter taste and acts as a repellent. After this training, the flies were scored to avoid the illuminated chamber; as the experiments were performed straight away, we could consider this as a measure of their learning ability. The experiments were repeated after five minutes, and in this case it would be an assessment of short-term memory. Although around 40% of control flies learned to avoid the light, only half this proportion of the *para^KO^* flies did the same (Figure 2D’), and the difference was statistically significant (*** *p* < 0.001). In the second experiment, fewer flies remembered to avoid the quinine, and the *para^KO^* flies also performed worse than the control (* *p* < 0.05). The reduction in number of flies remembering if avoid the light was similar within both genotypes, but this reduction was not statistically significant. Therefore, we can conclude that *para^KO^* flies have an impairment in their learning ability, but it is not that clear that they have a worse short-term memory.

### 3.3. The para^KO^ Larvae Have Electrophysiological Defects

**Figure 3:**
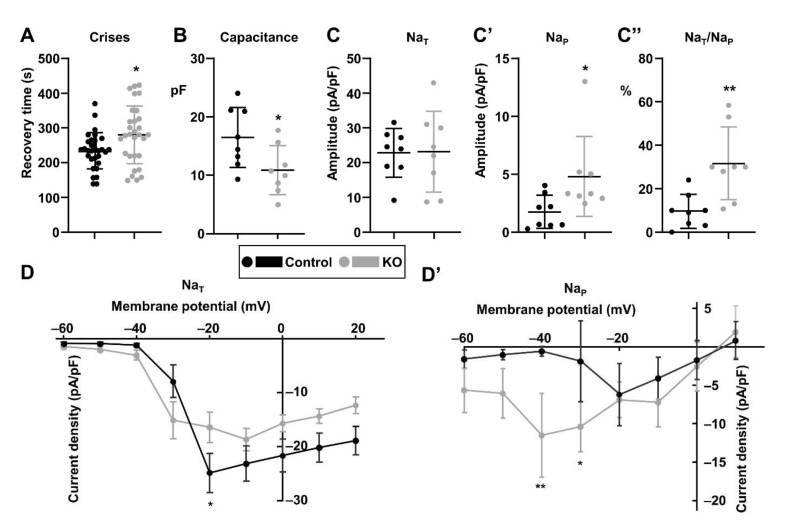
Electrophysiological analyses. (**A**). Recovery time of third instar larvae after electroshock. (**B**). Capacitance of the aCC neurons in which the sodium currents were measured. (**C**–**C’’**). Analysis of sodium currents in voltage clamp experiments at a fixed membrane potential: amplitude of transient sodium currents (**C**), persistent sodium currents (**C’**), and the ratio of the amplitudes of transient to persistent sodium currents (**C’’**). (**D**,**D’**) Variation of the amplitude of transient (**D**) and persistent (**D’**) sodium currents at different membrane potentials. (A–C), Student’s *t*-test; (**C’**,**C’’**) Mann–Whitney; (**D**,**D’**), two-way ANOVA followed by Bonferroni’s multiple comparisons test. Bars represent mean ± SD (* *p* < 0.05; ** *p* < 0.01).

Our previous results showed that the *para* knock out suffered temperature-induced seizures, most probably due to the alteration of sodium currents. In order to characterise these currents we decided to carry out patch clamp electrophysiological analyses of the mutant neurons. We performed these studies using the well characterised aCC motor neurons in late third instar larvae (120 h after egg laying), just before puparium formation. These neurons are easy to manipulate and identifiable with the use of fluorescent genetically-engineered probes (Baines et al., 2017). In order to find out if larvae also had a convulsive genotype, we used a well-established assay, electroshock-induced seizures. When the electroshock is applied with a metal probe, the larvae suffer spasms and tonic contractions for a period of time, and finally recover and resume their normal ambulation pattern. The *para^KO^* larvae had a significantly longer recovery time (Figure 3A), demonstrating that the seizure susceptibility is already established at this developmental stage.

In order to identify possible defects in sodium currents, we performed voltage clamp, to analyse the sodium transient and persistent currents (Na_T_ and Na_P_, respectively). As their name indicates, Na_T_ is a brief, but large, current that supports the upstroke of action potentials. This current component is transient due to internal block of the ion pore. By contrast, Na_P_, which represent random reopenings of inactivated channels, is smaller in amplitude but longer in duration. A first observation was that in the dissected larval brains the aCC neurons seemed to be smaller. One of the parameters obtained from the patch clamp assays is the capacitance of the clamped cell, and this is directly proportional to the cell size. aCC neurons in the *para^KO^* brains had a lower capacitance than wild type neurons (Figure 3B), so we can conclude that the mutant neurons are indeed smaller.

Regarding the amplitude of the sodium currents, Na_T_ did not show any significant changes (Figure 3C), while *para^KO^* cells had an increased Na_P_ (Figure 3C’); and consequently also an increased Na_P_/Na_T_ ratio (Figure 3C’’). An increase in this ratio is a defining feature of *Drosophila* seizure mutants, and is corrected in anticonvulsant treatments [19,25,43].

In addition to their amplitude at a fixed membrane potential, we analysed the current: voltage relationship for each current component, to shed light on the kinetics of the sodium channels. Regarding the initial Na_T_, in both genotypes Na entry started at a similar membrane potential, but they reached their maximum intensity at different potentials, −20 mV for wild type neurons and at −10 mV for *para^KO^* neurons (Figure 3D). Therefore, in the mutant genotype the channel activation was delayed compared to the wild type. The trend of the Na_P_ was the opposite (Figure 3D’): *para^KO^* neurons started with increased Na_p_ currents from higher membrane potentials, and this difference was most significant at potentials of −40 and −30 mV, when the mutant neurons reached their peak. In contrast, in wild type neurons the highest current was reached at −20 mV. In summary, in *para^KO^* neurons Na_P_ started at higher membrane potential and had an increased amplitude. This would mean that this current component is more easily activated in *para^KO^* neurons than in wild type.

The elevated Na_P_ persistent sodium currents, are a common feature in epilepsy, including *SCN1A*-related syndromes, and therefore targeting them is an interesting therapeutic strategy [44,45].

### 3.4. The Metabolome of para^KO^ Fly Heads

During the experiments to evaluate the learning and memory of the *para^KO^* flies we realised that they had an increased mortality rate during the previous fasting period, which pointed towards a metabolic alteration. This result was somewhat surprising, since in normal conditions both genotypes have no differences regarding viability or spontaneous seizures. However, in this case the flies are subjected to a severe 16-h starvation to make them more active in the search for food. It is only in these strenuous situation that the underlying metabolic alterations are evident.

Recent work *Drosophila para* mutants aimed at understanding dietary interventions, such as the ketogenic diet have revealed that certain food compositions can alleviate the symptoms [46,47]. This evidence led us to carry out a metabolomic analysis to determine if there were differences in basal metabolite levels between the *para^KO^* and the control flies. Since our focus was in neuronal phenotypes, we performed this analysis in adult head extracts, in which the neural tissue is over-represented in comparison with other anatomical regions.

From each head homogenate, we separated the aqueous and organic phases, to obtain information about the metabolome and the lipidome. ^1^H NMR spectra were acquired for each phase, and the main signals identified and quantified. Visual inspection indicated some anormal, large signals in samples DH2 and DH4, probably coming from contaminations during the extraction process. For this reason we decided to exclude this samples from the analysis, which would still leave five samples per genotype, so we did not consider it necessary to include new replicates.

**Figure 4:**
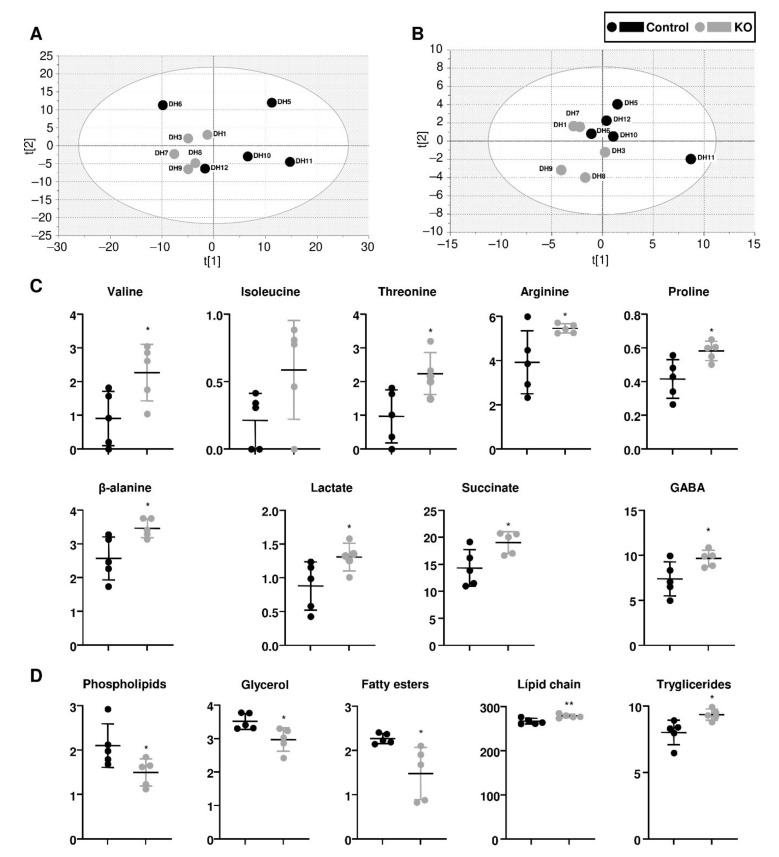
Metabolomic analysis. (**A**) PCA score plot of ^1^H NMR spectra of aqueous head extracts, samples DH2 and DH4 were excluded. UV scaling. R2X(cum) = 0.74, Q2(cum) = 0.31. 3 component model. (**B**). PCA score plot of ^1^H NMR spectra of organic head extracts o. UV scaling. R2X(cum) = 0.73, Q2(cum) = 0.28. 2 component model. (**C**) Relative abundance of metabolites in the aqueous extracts that showed significant differences. (**D**) Relative abundance of metabolites in the organic extracts that showed significant differences. In (**C**,**D**), relative abundance is calculated as concentration normalised to the total area of the spectrum. In all the experiments Student’s *t*-test was used (* *p* < 0.05, ** *p* < 0.01).

Principal component analyses indicated that there was a separation of the genotypes in both types of extracts (Figure 4A,B), indicating differences in the metabolic profile. Next, by performing a univariate statistical analysis, we identified those metabolites that had the most significant differences. In the aqueous extracts (Figure 4C), we found increased levels for several amino acids: valine, isoleucine, threonine, arginine, proline, and β-alanine. In addition, there were increased levels of lactate and succinate, which was indicative of a mitochondrial dysfunction and an increase in oxidative stress.

Although amino acid imbalance has been reported in epileptic patients, the patterns of abundance are not consistent and may vary depending on the particular form of the disease [48]. In contrast, metabolic imbalance and increased oxidative stress are common hallmarks in different types of epilepsy [8,49]. The ketogenic diet, an intervention that is being used in DS, can impact of both the amino acid imbalance and the neuroinflammation caused by oxidative stress [50,51]. Some of these alterations have been already described in DS models and patients, such as the increase in lactate in the serum of patients, or the increase in succinate in zebrafish models [10,52]. Oxidative stress is linked to increased neuroinflammation in epilepsy [9,53], and this has also been found in DS models and patients [40,54].

The aqueous extracts of the *para^KO^* heads also had increased levels of the GABA neurotransmitter. Since DS affects primarily gabaergic neurons, it is tempting to speculate that this represents an accumulation of neurotransmitter due to the lack of activity of the neuron, but this possibility would require further investigation.

Regarding the organic extracts, there were differences in some lipids, such as decreased levels of phospholipids and fatty esters, or slightly increased levels of lipid chain and triglycerides (Figure 4D). The increase in triglycerides, in combination with a reduction in phospholipids and linear fatty esters (membrane components), is an indicator for the accumulation of lipids in the form of lipid droplet inside the cells. This is further confirmed by the reduction in the aqueous metabolite glycerol, which is required for triglyceride synthesis. Lipid droplets are cellular fuel stores, signalling hubs, protective waste reservoirs for hyperactive neurons, products of lysosomal dysregulation, and hallmarks of ageing [55]. Within the nervous system, lipid droplets are associated with several pathologies including amyotrophic lateral sclerosis, and Alzheimer’s, Huntington’s and Parkinson’s disease, and can be correlated with increased oxidative stress and inflammation [56,57,58]. Therefore, the lipidomics of the head extracts would also be consistent with neuroinflammation in the *para^KO^* flies.

## 4. Conclusions

In this work, we describe the generation and characterisation of a knock-out allele in the *para* gene of *Drosophila*, as a tool for research and therapeutic development in DS. In the new mutant, gene expression was completely abolished, so it is a null allele. Regarding viability, the new allele is a recessive lethal, and the period of lethality has been established as the end of embryonic development, without any evident anatomical defects. Genetically, it is a haplo-insufficient dominant allele regarding seizures and other neurological phenotypes.

The additional neuromuscular, behavioural, and learning defects are highly reminiscent of those present in DS patients. The heterozygote *para^KO^* flies also display electrophysiological alterations similar to those present in DS and other forms of epilepsies and sodium channelopathies, mainly an increase in persistent sodium currents, and a defective nerve conduction. The heterozygote *para^KO^* flies have a metabolomic profile consistent with metabolic imbalance, mitochondrial dysfunction, increased oxidative stress, and neuroinflammation.

In summary, the genetics, neurobiology, and physiology of this new model is representative of many of the traits present in DS patients. Therefore, it may constitute a useful tool to investigate the pathophysiology of the disease and to search for new treatments, biomarkers or genetic modifiers.

## Data Availability

The NMR metabolomics data that support the findings of this study are openly available in Zenodo at https://doi.org/10.5281/zenodo.5658659, uploaded 9 November 2021.

## Appendix A

**Table A1:** **Primers.** Restriction enzyme target sequences are underlined.

Use	Primer Sequence	Direction
**Generation of the A arm of homology**	AGAGGCTAGCCAGTATTCGGGCAACTGCTG	Forward
	AGAGAGGTACCTGTCTATGCGGCCAACGATGA	Reverse
**Generation of the B arm of homology**	AGAGAACTAGTTCCGACTCGATATCTGAGGAAG	Forward
	GTGGACATATGCTGTTCTAGT	Reverse
**Validation of the A arm recombination**	TCGCTGGTGGTAG	Forward
	CGAAGTTATGGTACACT	Reverse
**Validation of the B arm recombination**	ATAGGAAGTTATCACTAGTTCCG	Forward
	GACAGAATAGACAGTATGCAG	Reverse
***para*** **qPCR**	CTGAACATGAAAAGCAGAAGG	Forward
	CCTGTTCAAGTGTAGGATCCG	Reverse
***rp49*** **qPCR (control)**	CGTTTACTGCGGCGAGAT	Forward
	GCGCTCGACAATCTCCTT	Reverse
**Validation of the excision by PCR**	CGAAGATGAGGAAGACACAT	Forward
	GACAGAATAGACAGTATGCAG	Reverse

**Table A2:** ***Drosophila* Stocks**. B# indicates Bloomington *Drosophila* Stock Centre stock number.

Stock	Origin
***white^1118^***	B#3605
***Canton-S***	B#64349
***Ubiquitin-Gal4^3xP3-GFP^***	Luis Alberto Baena-Lopez
*hs-Cre*; *TM2*/*TM6B*	B#6936
y^1^ w*/*Dp(2;Y)^G^*, P{w^+mC^ = hs-hid}^Y^; P{ry^+t7.2^ = 70FLP}^23^ P{v^+t1.8^ = 70I-SceI}^4A^/TM3, P{w^+mC^ = hs-hid}^14^, Sb^1^	B#25679
***para^KO^*** **/*FM7c-GFP***	This work
*Df(1)FDD-0230908, w^1118^/FM7c*	B#23296
***para^bss1^***	Mark Tanouye
